# Spironolactone Versus Oral Contraceptive Pills in the Treatment of Adolescent Polycystic Ovarian Syndrome: A Systematic Review

**DOI:** 10.7759/cureus.25340

**Published:** 2022-05-25

**Authors:** Shriya Rajashekar, Suganya Giri Ravindran, Meghana Kakarla, Musa Ausaja Gambo, Mustafa Yousri Salama, Nathalie Haidar Ismail, Pardis Tavalla, Pulkita Uppal, Shaza A Mohammed, Pousette Hamid

**Affiliations:** 1 Internal Medicine, California Institute of Behavioral Neurosciences & Psychology, Fairfield, USA; 2 Research, California Institute of Behavioral Neurosciences & Psychology, Fairfield, USA; 3 Neurology, California Institute of Behavioral Neurosciences & Psychology, Fairfield, USA

**Keywords:** oral contraceptive pills, combined oral contraceptive pills, spiomet, spironolactone, coc, ocp, adolescent pcos, adolescent, pcos, polycystic ovarian syndrome

## Abstract

Polycystic ovarian syndrome (PCOS) is a multi-system endocrinopathy that affects women of reproductive age. Due to features that coincide with puberty, it frequently remains undiagnosed in adolescent females. The lack of evidence on management alternatives has resulted in significant variation in practice. This systematic review evaluated the therapeutic advantages and adverse effects of a regularly used therapy option, combined oral contraceptive pills (COC/OCP) with spironolactone (SP), a newer alternative that may be used alone or in conjunction with other drugs to treat adolescent PCOS. A literature search was conducted using PubMed, PubMed Central, Scopus, and Google Scholar. It was restricted to studies published in English between 2021 and 2011 that discussed the management of adolescent PCOS with COC, SP, or both. The systematic review followed the Preferred Reporting Items for Systematic Reviews and Meta-Analyses 2020 guidelines. Two reviewers independently examined the content of the included studies using appropriate quality assessment tools. Four meta-analyses, four randomized controlled trials (RCTs), and one traditional review were found to be eligible. After extensive analysis, we concluded that SP, alone or in combination, is far safer than COC. However, COC treats more PCOS-associated symptoms than SP, including acne and menstrual irregularities, while also providing contraceptive benefits. However, SP monotherapy is cardioprotective and therapeutic when combined with other drugs. Long-term COC use has been linked to an increased risk of venous thromboembolism, hypertension, dyslipidemia, low-density lipoprotein (LDL) elevation, dysglycemia, and cancer in women.

## Introduction and background

Polycystic ovary syndrome (PCOS) is defined by a combination of signs and symptoms of androgen excess and ovarian dysfunction in the absence of other specific diagnoses [1]. It is a heterogeneous, multi-system endocrinopathy that manifests in the ovary and presents with a range of clinical characteristics, the three most common of which are menstrual abnormalities, elevated androgen levels, and cystic ovaries [2,3]. As a tribute to the two physicians who first identified it in 1935, the disease is recognized as Stein Leventhal Syndrome also [2].

The current global prevalence of PCOS, according to National Institutes of Health (NIH) standards, is 6% (5%-8%); however, it is 10% when Rotterdam or the androgen excess and PCOS society (AE-PCOS Society) criteria are used [4]. It is one of the most common endocrine disorders among reproductive-age women in the United States, affecting 5%-10% of the fertile female population, 4%-8% of whom are young adults and middle-aged women [4]. PCOS is more common in African American women (8.0%) than in white women (4.8%), with a total prevalence of 6.6% (difference not statistically significant, P > 0.05) [5].

It is a result of strong epigenetic and environmental influences, altering lifestyles, stress, and diets [1]. It's also becoming increasingly common in adolescent females, presenting shortly after puberty.

Making a clinical diagnosis is especially difficult due to widespread pubertal overlap symptoms, and because there is no criteria for identifying PCOS in adolescent females, it frequently goes undiagnosed [6].

PCOS also has long-term consequences, including an increased risk of atherosclerotic disease, hypertension as well as impaired glucose tolerance. Childhood development of type 2 diabetes, which girls with adolescent PCOS are more vulnerable later in adulthood [7-9].

The current standard of care for adolescent PCOS is a blend of lifestyle interventions, combined oral contraceptives (COC) (oestrogen and progesterone preparations), metformin, and anti-androgens such as flutamide, finasteride, or spironolactone (SP), either alone or in conjunction [6,10-14]. COC is often used but also despised due to the stringent adherence required and the unpleasant and worrisome side effects such as breast tenderness, bloating, nausea, weight gain, elevated blood sugars, dyslipidemia, venous thromboembolism, cardiovascular disease, irregular bleeding, and hypertension [15]. A recent study suggests that early COC exposure may raise the risk of cancer [9,16].

Spironolactone, alone or in combination, has emerged as a promising therapeutic alternative to COC. Spironolactone is an anti-androgenic medication with several additional advantages in the treatment of women with PCOS. Additionally, it has a broad safety profile with lesser side effects that are more acceptable [17-19].

The objective is to address the PICO question, with the population of interest being adolescent females with PCOS because it is vital to treat young girls with efficacious and safe medications that will not impair their future life. The goal of this intervention is to compare COC and Spironolactone, two commonly used drugs. The outcome will include a determination of the effectiveness and side effects of the two medications mentioned.

**Figure 1:**
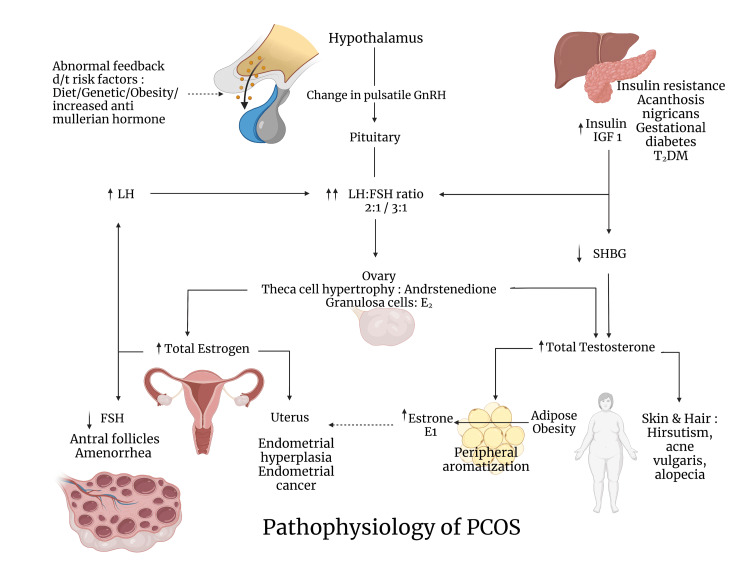
Pathophysiology of PCOS FSH: follicular stimulating hormone; LH: luteinising hormone; SHBG: sex hormone-binding globulin; T2DM: type 2 diabetes mellitus; E1: estrone; E2: estradiol; IGF1: insulin-like growth factor 1; PCOS: polycystic ovarian syndrome. Created with BioRender.com by the authors.

Figure 1 depicts the pathophysiological events that lead to various clinical features in PCOS.

## Review

Methodology

We conducted a systematic review of freely available full-length articles and utilized the Preferred Reporting Items checklist to document our methodology and conclusions. We have adhered to the PRISMA 2020 recommendations [20-22]. 

Search Strategy

Using the preferred reporting items for systematic reviews and meta-analyses (PRISMA) statements and flowcharts, the studies were organized for synthesis [20,22]. This allows for a more systematic search and identification of research to include in the review. Electronic searches on PubMed, PubMed Central, Scopus, and Google Scholar were undertaken in December 2021. A Boolean search was conducted utilizing MeSH, regular keywords, and synonyms relating to the topic at hand, including "Polycystic ovarian syndrome," "Spironolactone," and "Combined Oral Contraceptives," both alone and in combination. The search also used the built-in search functions of the websites to aid in the selection process. Following this, all articles were retrieved, and only those articles relevant to the study were included.

Inclusion Criteria

Inclusion criteria were (i) only full-text peer-reviewed articles, as well as (ii) all study designs that were published between 2021 and 2011. (iii) Articles written in English, (iv) with the availability of an abstract, and (v) the full, unrestricted text focused on (vi) adolescent females between the ages of 11 and 19 were chosen. The term "adolescent" refers to the age group established by the World Health Organisation (WHO) [23]. (vii) Studies with a broader age range of data, including children and adolescents, separately were deemed eligible.

(viii) Individuals suffering from PCOS as defined by the NIH, Rotterdam, or AE-PCOS Society criteria [24,25], who received (ix) treatment given with COC alone or in combination; (x) Spironolactone used alone or in combination with other pharmaceuticals. (xi) Demography incorporated research from all over the world.

Exclusion Criteria

The review excludes studies of (i) unpublished articles, case reports, grey literature, and research written before 2011, (ii) Studies without access to the full text, (iii) non-female gender, and (iv) if the study's age does not include an adolescent population. (v) Other pediatric endocrinopathies causing similar symptoms are excluded. (vi) Management of PCOS without the use of Spironolactone or/and COC; (vii) Studies that include fertility induction medication and fertility as an outcome measure (viii) as well as those with a low-quality appraisal of the eligibility papers [20].

Data Extraction

Two independent reviewers undertook the selection procedure. The first step (i.e., identification) included developing search queries for PubMed, PubMed Central, Scopus, and Google Scholar and identifying records. Having followed that, records designated as ineligible were deleted using the database's automated tools. Later, using the EndNote program, duplicates were deleted. The second step (i.e., screening) included reading titles and abstracts of articles and selecting those that could be relevant based on the inclusion and exclusion criteria. Following that, manual search and EndNote were used to locate the full text of the reports. We examined 1048 records and identified 59 articles for full-text screening, eligibility, and quality assessment.

Quality Assessment and Analysis of the Studies

We assessed the quality of articles throughout the eligibility phase utilizing quality assessment measurement tools. (i.e., phase 3). For Systematic Reviews & Meta-Analysis, the assessment of multiple systematic reviews (AMSTAR 2) checklist was used for Traditional Reviews, and the scale for the assessment of narrative review articles, SANRA Checklist, was implemented [26,27]. Cochrane risk of bias, version 29, was utilized for the quality assessment of randomized control trials [28]. After the assessment of eligibility, the articles where both reviewers had a 95% accord have been included. Disagreements were addressed by discussion between the other authors and, if required, consultation with our mentor as well as an external reviewer. This review has a total of nine articles.

Table 1 summarizes the baseline characteristics of studies that have been included.

**Table 1:** Table of data extraction A summary of the included studies. RCT: randomised control trials; BMI: body mass index; COS: cross-over study; COC: combined oral contraceptives; SP: spironolactone; SPIOMET: low-dose combination of spironolactone, pioglitazone, and metformin; HMW- adiponectin: high-molecular-weight adiponectin.

Author	Year of publication	Study design	Quality appraisal tool	Primary studies	Outcome measure
Hall et al. [29]	2012	RCT	Cochrane risk of bias assessment tool	At the start of a six-month COC intervention, 354 young women received standard psychological assessments and were questioned about COC side effects and use. The associations between psychological circumstances perceived COC side effects, and continuation rates were examined using logistic regression.	Depressed mood, stress and perceived weight change.
Domecq et al. [30]	2013	Meta-analysis	AMSTAR 2 checklist	20 RCTs, one prospective cohort, one case control, two COS.	Lactic acidosis, thromboembolic episodes, liver toxicity, cancer incidence, pregnancy loss, weight gain and cardiovascular disease.
Alpanes et al. [31]	2017	RCT	Cochrane risk of bias assessment tool	A randomised, parallel, open-label clinical research for 12 months, with 92 patients comparing COC with SP to metformin in women with PCOS.	Amelioration of hirsutism, androgen excess, menstrual dysfunction and cardio-metabolic safety (changes in the frequencies of disorders of glucose tolerance, dyslipidemia, and hypertension).
Liu et al. [32]	2017	Meta-analysis	AMSTAR 2 checklist	19 cross-sectional, one case control, four cohort with 270,284 participants	Hypertension
Diaz et al. [33]	2018	RCT	Cochrane risk of bias assessment tool	Over 12 months, 35 PCOS females were randomly assigned to receive COC (n = 18) or SPIOMET (n = 17). Healthy adolescent girls were used as controls (n = 25).	Fetuin-A and hepatic visceral fat.
Alalami et al. [34]	2019	Literature review	SANRA checklist	Nine literature reviews, one meta analysis, seven RCTs, one prospective open label study, one multi-centric study, one prospective cohort study.	Cardiovascular profile evaluations are connected with the drugs utilised in PCOS management.
Almalki et al. [35]	2020	Meta-analysis	AMSTAR 2 checklist	Nine RCT's comprising 613 patients were included.	Testosterone levels.
Ibanez et al. [36]	2020	RCT	Cochrane risk of bias assessment tool	Two pilot investigations examined the effects of randomised therapy with an oral COC or SPIOMET in non-obese girls with PCOS (n = 62).	On-and post-treatment, fasting insulin, androgens, HMW adiponectin, and microRNA [miR]-451a were assessed. Body composition and hepato-visceral fat were also assessed.
Al Khalifah et al. [37]	2021	Meta-analysis	AMSTAR 2 checklist	37 RCTs comprising 2400 patients were included.	Menstrual regulation and change in hirsutism scores are the primary outcome measures. The secondary outcome variables are acne scores, the prevalence of dysglycemia, BMI, lipid profile, total testosterone level, and any problems that might happen.

**Figure 2:**
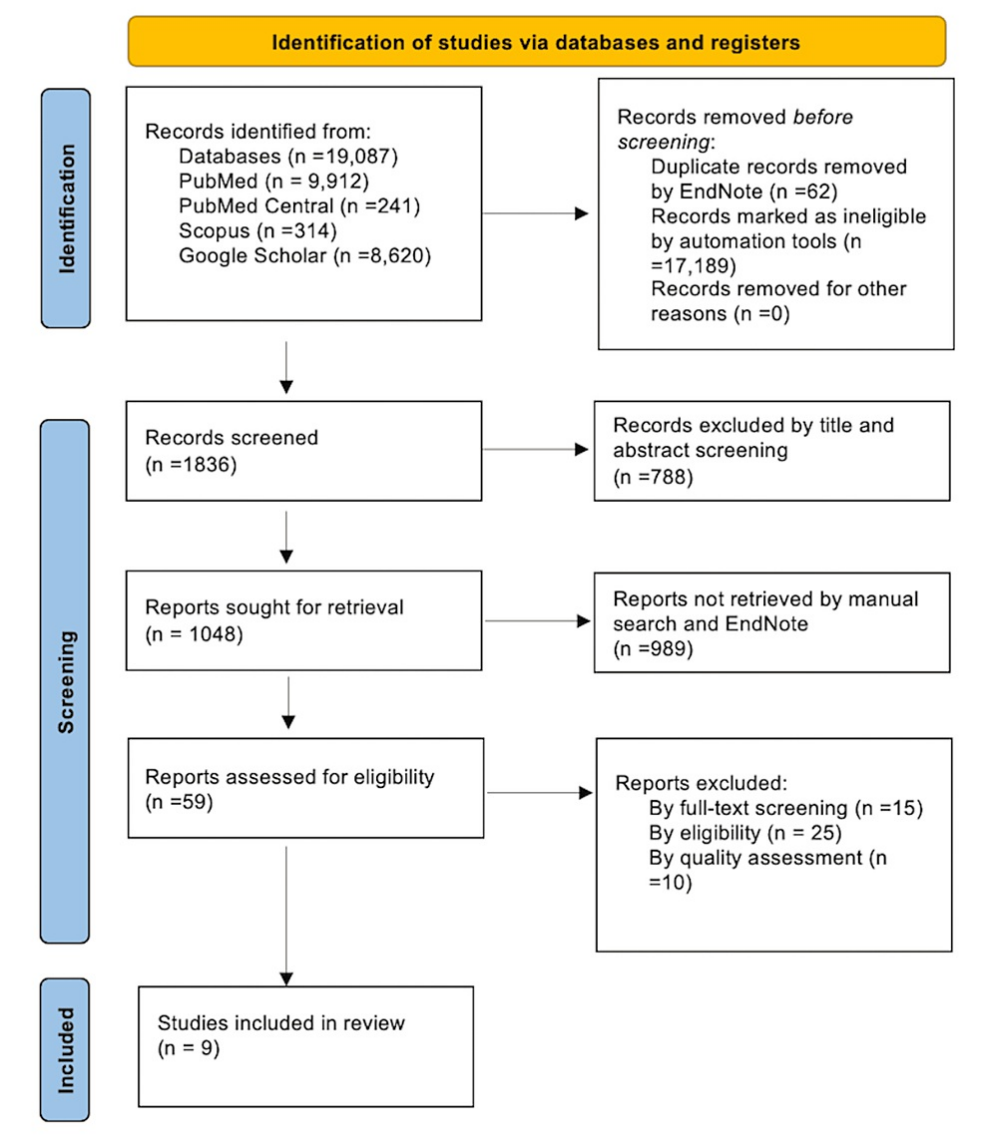
PRISMA 2020 flow diagram PRISMA: Preferred reporting items for systematic reviews and meta-analysis. The figure was created by the authors.

A comprehensive PRISMA flow chart is depicted in Figure 2 [22].

The keywords utilised in the study are depicted in Table 2.

**Table 2:** Keywords employed in the study The search was undertaken in December 2021. MeSH: medical subject headings.

Search strategy	Keywords
Regular keywords	Polycystic ovary syndrome; PCOS; stein-leventhal syndrome; oral contraceptives; combined oral contraceptive; COC; OCP; Spironolactone; SP; Aldactone; Adolescent
MeSH keywords	(( "Polycystic Ovary Syndrome/drug therapy"[Mesh] OR "Polycystic Ovary Syndrome/therapy"[Mesh] )) OR ( "Polycystic Ovary Syndrome/drug therapy"[Mesh:NoExp] OR "Polycystic Ovary Syndrome/therapy"[Mesh:NoExp] ) AND (( "Spironolactone/adverse effects"[Majr] OR "Spironolactone/pharmacokinetics"[Majr] OR "Spironolactone/therapeutic use"[Majr] OR "Spironolactone/toxicity"[Majr] )) OR ( "Spironolactone/adverse effects"[Mesh:NoExp] OR "Spironolactone/pharmacokinetics"[Mesh:NoExp] OR "Spironolactone/therapeutic use"[Mesh:NoExp] OR "Spironolactone/toxicity"[Mesh:NoExp] ) AND (( "Contraceptives, Oral/adverse effects"[Majr] OR "Contraceptives, Oral/pharmacokinetics"[Majr] OR "Contraceptives, Oral/therapeutic use"[Majr] OR "Contraceptives, Oral/therapy"[Majr] OR "Contraceptives, Oral/toxicity"[Majr] )) OR ( "Contraceptives, Oral/adverse effects"[Mesh:NoExp] OR "Contraceptives, Oral/pharmacokinetics"[Mesh:NoExp] OR "Contraceptives, Oral/therapeutic use"[Mesh:NoExp] OR "Contraceptives, Oral/therapy"[Mesh:NoExp] OR "Contraceptives, Oral/toxicity"[Mesh:NoExp] ) AND adolescent

Results

Literature Search

The search yielded 1048 possibly relevant articles, 59 of which had full-text access. The papers were then scrutinized for eligibility, with 25 being rejected owing to noncompliance with the criteria. During data extraction and full-text screening, another 15 papers were excluded from the research. Finally, we used quality assessment methods to examine publications before incorporating them in the systematic review, and 10 papers were found to be ineligible. In the end, nine papers were approved.

Study Characteristics

The systematic review includes four meta-analyses, four RCTs, and one traditional review. In all of the articles chosen, combined oral contraceptive pills (COC) or Spironolactone (SP) have been an intervention for treating adolescent polycystic ovarian syndrome (PCOS). Two studies directly assessed the efficacy of all treatment methods for adolescent PCOS. One study examined the risks and side effects of all PCOS medications. One study evaluated the cardiovascular characteristics of medications used to treat adolescent PCOS. Two trials examined the combination therapy for SP, while one examined the combination therapy for COC. Two studies studied the long-term effects of COC and compared the outcomes of patients who take COC vs. those who do not. It is critical to highlight that there is presently no highly effective treatment for PCOS. Due to the complexity of the syndrome in its entirety, each symptom may be addressed individually with a tailored specific strategy.

Clinical Efficacy

Menstrual regulation: was observed to be better with COC plus spironolactone (OR: 0.06, 95% CI: 0.02-0.23) compared to metformin alone, according to an RCT by Alpanes et al. [31]. In addition, Ibanez et al. found that SPIOMET an abbreviation for a low-dose combination of spironolactone 50 mg/day, pioglitazone 7.5 mg/day, and metformin 850 mg/day, made the menstrual cycle more regular in 90% of adolescents with PCOS [36]. This was compared to COC (20μg ethinyl estradiol plus 100 mg levonorgestrel), which was seen as effective only in 42% of the adolescents. There are no statistically significant benefits to using SP in menstruation regulation [37].

Dysglycemia: Domecq et al. reported that COCs had no net effect on fasting blood glucose (WMD1.18, 95% CI6.99 to 4.63; P=0.69), and there was no evidence of the incidence of Type 2 Diabetes [30]. According to Alpanes et al., a combination of COC and Spironolactone had no effect on dysglycemia as determined by fasting insulin (89 ± 97), HOMA-IR (1.6 ±1.7), and insulin sensitivity index (6.7 ± 5.1) [31]. It was compared to metformin, a hypoglycaemic drug that improved fasting insulin levels (69 ±51), HOMA-IR (1.3 ±0.9), and insulin sensitivity index (7 ± 5.5). Ibanez et al. stated that after one year, insulin resistance (IR), as evaluated by the HOMA-IR level, decreased significantly in the group using SPIOMET(1.2 ± 0.1) compared to the group taking COC (Ethinyl estradiol-Levonorgestrel) (3 ±0.3) [36]. This demonstrates that a low-dose spironolactone combination (SPIOMET) is more effective than COC at reducing dysglycemia. Al Khalifah et al. reported dysglycemia outcomes were available for seven therapies across 10 RCTs (n = 639) [37]. The absolute incidence of dysglycemia was observed to be 57 per 100 patients among COC users (95 % [CI], 1-100 more patients), compared to a baseline risk of 24% among controls. There was no statistically significant difference in dysglycemia on using Spironolactone [37].

Testosterone: According to an RCT conducted by Alpanes et al., with 46 PCOS women, including an adolescent population, 24 women were assigned to COC plus spironolactone, a combination of an oral contraceptive pill containing 30 mcg ethinyl estradiol and 150 mcg desogestrel and 100 mg/day of SP, and 22 women were assigned to metformin, 850 mg twice daily [31]. The total testosterone (1.1 nmol/L,0.4-1.7), free testosterone (25 pmol/L,12-39), and androstenedione (5.5 nmol/L,1.8-9.2), dehydroepiandrosterone sulphate (2.7 μmol/L,1.4-4.0) were decreased significantly in this study [31]. In a meta-analysis published in 2020 by Almalki et al., it was discovered that SP (MD−2.90, 95%CI−3.77,−2.02) and spironolactone metformin combination (MD−2.83, 95%CrI−3.80,−1.87) reduced testosterone levels more than COC alone (MD−2.78, 95%CrI−3.60,−1.97) [35]. In a 12-month RCT conducted by Ibanez et al. ( n = 62, mean age = 15.8 years), there was a substantial drop in free testosterone levels in both SPIOMET (0.8 ± 0.1) and COC (0.7 ± 0.1) [36]. Almalki et al. observed that COC (MD-2.78, 95 percent CI -3.60, -1.97), spironolactone with metformin (MD2.83, 95 percent CI 3.80, 1.87), and spironolactone alone (MD-2.90, 95 percent CI -3.77, -2.02) all reduced free testosterone levels [35]. COC had a greater reduction in free testosterone levels than spironolactone alone or in combination. According to the data provided by Al Khalifah et al. on free testosterone levels, treatments with spironolactone and COC were assessed to lower total testosterone levels relative to placebo in 34 studies (n = 1811) [37].

Lipid profile: Alpanes et al. showed no difference in dyslipidaemia (OR: 0.6, 95% CI: 0.2-1.8) in an RCT (n=46) comparing a combination of COC + Spironolactone and Metformin [31].

Table 3 summarises the effects of SP, SIPOMET, and COC on lipid profile. Referenced from Ibanez et al. and Al Khalifah et al. [36,37].

**Table 3:** Summary of lipid profile HDL: high density lipoprotein; LDL: low density lipoprotein; SPIOMET: low dose spironolactone with pioglitazone and metformin; COC: combined oral contraceptives.

Lipid profile	Possible impact
Total cholesterol	COC mono-therapy resulted in a significant rise in total cholesterol levels.
Triglyceride	The rise in triglycerides was seen with COC compared to the control group. It was lower with SPIOMET during 12 months. In a recent study, it was observed that there were no statistically significant changes in treatment with COC or spironolactone alone or combination with other medications.
LDL	LDL cholesterol increased with COC, and no change was detected with SPIOMET after 12 months, but a reduction was observed after 24 months with SPIOMET. Patients treated with metformin-flutamide combination therapy had a significant reduction when compared to COC mono-therapy.
HDL	HDL increased with COC after 24 months but was statistically insignificant. No change was detected in the SPIOMET users. Spironolactone increased in HDL.

Hirsutism: In an RCT (n=46), Alpanes et al. assigned 24 patients to COC (30 mcg ethinyl estradiol plus 150 mcg desogestrel) plus spironolactone(100 mg/day) and 22 patients to metformin, which was started at 425 mg and maintained at an 850 mg [31]. It was found that COC plus spironolactone caused a reduction in hirsutism score than metformin (MD 4.6, 95% CI: 2.6-6.7). According to Ibanez et al., the hirsutism score dropped significantly in the SPIOMET (11± 1) compared to the COC (14 ± 1) after 12 months [36]. In a meta-analysis by Al Khalifah et al. using the Ferriman-Gallwey scoring system, 13 of 25 trials (n = 1401) reported hirsutism. Spironolactone or metformin-spironolactone combination treatment is linked with a statistically significant decrease in hirsutism when compared to placebo. All combination medications, however, resulted in a 2.5-point reduction in hirsutism score when compared to mono-therapy. Indicating that combination medicines are superior to mono-therapies [37]. COC exhibited statistically significant reductions in hirsutism scores as compared to placebo, progesterone (MD, -5.66; 95% [CrI], -8.97 to -2.46), cyproterone acetate (MD, -3.06; 95% CrI, -5.05 to -1.02), drospirenone (MD, -3.00; 95% CrI, -5.52 to -0.48) and desogestrel (MD, -3.16; 95% CrI, -5.29 to -0.9). No statistically significant improvements with norgestimate or gestodene were seen [37].

Acne: The type of progesterone contained in the COC appears to have an effect on acne. Cyproterone acetate and chlormadinone acetate are superior to COC containing levonorgestrel [38]. Due to a lack of reliable research and the lack of a validated scale for evaluating acne, the outcome is mixed, with slight favor for COC in terms of acne reduction by MD 0.5 to 2 from baseline [37].

BMI & weight: Domecq et al. reported that COC (Ethinyl-estradiol 30mcg + Chlormadinone acetate 2 mg and Ethinyl-estradiol 30mcg + Drosperinone 3 mg) had no effect on BMI (COCs: BMI WMD -0.001, 95% CI- 0.16 to 0.16,P= 0.99, I ²= 0) [30]. In one trial, Domecq et al. found that COCs had no effect on weight gain (COCs: WMD 0.04, 95%CI -0.35 to 0.43,P= 0.84) [30]. Ibanez et al. concluded that after one year, BMI rose with COC ( D0-12 mth = 0.7± 0.3) compared to those receiving SPIOMET (D0-12 mth = -0.2 ± 0.3), these findings, however, are statistically significant (P < 0.05) [36]. Al Khalifah et al. examined 34 RCTs (n = 1798) and discovered no significant changes in weight loss or BMI treated with SP and COC [37].

Fetuin-A: Is a glycoprotein that is largely synthesized in the liver and is released in high amounts into the bloodstream in patients with fatty liver disease [33,39]. In humans, elevated fetuin-A levels have been linked to an increased risk of T2D and metabolic syndrome characteristics [33,40]. Contrary to expectations, increasing fetuin-A levels prevent vascular calcification and act as a protective factor during systemic inflammation [33,41]. Thirty-five PCOS adolescents were evaluated for a year in an RCT by Diaz et al.; they discovered that a low-dose combination of insulin sensitizers and SP, such as SPIOMET (1.13±0.05), raised fetuin-A levels as compared to COC users (0.94 ± 0.04) [33].

Side Effects

Venous thromboembolism (VTE): COCs were connected to a three- to six-fold increase in the relative risk of VTE in a WHO-led study [30]. VTE incidence increased from 5/10000 in never-users to 9-10/10000 in COC users, according to observational research involving 144,575 women-years of follow-up [30,42]. Lidegaard et al. research followed 1,626,158 non-pregnant women aged 15 to 49 years and discovered an increased risk of thrombotic stroke and myocardial infarction related to different COCs, with relative risks as high as 2.5 [42]. COCs containing Ethinylestradiol at 20 mcg increased the risk by a factor of 0.9-1.7, and at 30-40 mcg, the risk increased by a factor of 1.3-2.3 [30]. Third-generation COCs are associated with a higher incidence of VTE than second-generation COCs [30,43]. Additional risk factors, such as hypertension, obesity, diabetes, advancing age, smoking, and hypercholesterolemia, may enhance the risk of VTE [30,44].

Overall mortality and cardiovascular disease mortality: In a prospective cohort study with approximately 378,000 women-years that compared COC users to never-users, Domecq et al. reported a lower rate of mortality from any cause and a lower rate of death from cardiovascular illnesses [30,19]. Although recent studies have noted that long-term use of oestrogen-containing COCs may increase cardiovascular risk, it is uncertain if this translates into additional cardiovascular risk in young women with PCOS [34,45]. Spironolactone is useful in patients with documented cardiovascular disease and heart failure, according to Anand et al., spironolactone was demonstrated to lower triglycerides, elevate HDL, and lower IR in PCOS, as well as normalize endothelial dysfunction, although there were no cardiovascular risk benefits when combined with COC [17,34,46-48]. Spironolactone may thus have some cardioprotective advantage, although there are no medium-to-long-term clinical trials to advise on this in PCOS, even though it may be of clear benefit in heart failure patients [34].

Hypertension: According to Liu et al., there was a significant association between COC use and the risk of hypertension in women from Asia (RR, 2.12; 95% CI, 1.15-3.91), North America (RR, 1.32; 95% CI, 1.10-1.59), developed countries (RR, 1.26; 95% CI, 1.09-1.45), and developing countries (RR, 2.13; 95% CI, 1.06-4.27) [32].

The overall risk of cancer: The Hannaford et al. study examined the connection between COC consumption and cancer for 44 months [19]. When all malignancies were analyzed, it was discovered that women who took COC for more than eight years had a statistically significant higher chance of developing any cancer (adjusted relative risk 1.22, 1.07 to 1.39). Cervical cancers (adjusted relative risk 2.73, 1.61-4.61) and central nervous system or pituitary cancers (5.51, 1.38-22.05) were related to statistically significant elevated risks in long-term (8-year) users [19]. On the other hand, long-term oral contraceptive usage was related to a statistically significant reduction in the incidence of ovarian cancer (0.38, 0.16 to 0.88) and cancer of the uterine body [19].

Mood changes and compliance to treatment: Hall et al. observed that participants with a depressed mood at baseline were more than twice as likely to report expressed depressed mood and bodyweight changes at six months compared with those without a depressive state (OR 2.30, CI 1.30-4.07, p=0.004 and OR 2.14, CI 1.20-3.80,p=0.01) respectively [29]. Additionally, those who experienced stress were more than twice as likely to report "significant" mood changes as those who did not experience stress (OR 2.07, CI1.12-3.28, p=0.02) [29]. Additionally, Hall et al. discovered that adolescents who had perceived weight changes were 40% less likely to maintain COC than those who did not (OR 0.60, CI 0.38-0.94, p=0.03) [29]. 

Discussions

Efficacy of Spironolactone and COC

In our review, we observed that combined oral contraceptives (COC) in conjunction with Spironolactone (SP) or SPIOMET is considerably superior to COC alone in managing menstrual dysregulation in adolescents with PCOS. There was no high-quality evidence that SP alone could be used to treat menstrual dysregulation.

The absolute incidence of dysglycemia was observed to be 57% among COC users, compared to a baseline risk of 24% among controls. There was no evidence of the incidence of T2DM. A low-dose spironolactone combination (SPIOMET) proved more effective than COC at reducing dysglycemia outcomes.

Earlier studies found that spironolactone alone or in combination with metformin or pioglitazone and metformin or COC reduced serum testosterone levels more than COC alone.

However, in a research conducted by Almalki et al., COC reduced free testosterone levels more than spironolactone or spironolactone + metformin [35]. This debate was further explored in 2021 by Al Khalifah et al. on free testosterone levels, which concluded that spironolactone alone or in combination with other medicines is equally effective as COC in 34 studies (n = 1811) [37].

The use of COC alone resulted in a statistically significant increase in total cholesterol levels. In an RCT conducted by Ibanez et al., triglyceride levels were shown to be higher with COC and lower with SPIOMET [36]. However, in 2021, Al Khalifah et al. discovered no statistically significant changes in triglyceride levels in any of the two medications, COC or SP, or in their combinations, even when taken simultaneously [37]. High-quality evidence suggests that LDL levels rise after treatment with COC. This can be significantly observed after 12 months. Spironolactone had no significant correlation with LDL levels. SPIOMET, on the other hand, lowered LDL levels after 24 months. Evidence also suggests that HDL levels rise after spironolactone alone but not following SPIOMET.

Ibanez et al.'s evidence reveal an increase in serum HDL in COC users after 24 months, although it is not statistically significant [36].

Both COC and SP exhibit a decrease in hirsutism. However, when SPIOMET or COC was combined with spironolactone, a favorable reduction in hirsutism was noted when compared to COC alone. However, when contrasted to mono-therapy, all combination drugs resulted in a 2.5-point drop in hirsutism scores. This demonstrates the superiority of combination medicines over mono-therapies. The type of progesterone in COC has an effect on the degree of hirsutism. Progesterone, cyproterone acetate, desogestrel, and drospirenone all result in a favorable reduction. There were no statistically significant improvements in any comparison group when gestodene or norgestimate were used. The outcome for acne reduction in adolescent girls is mixed, with slight favor for COC.

The majority of research has shown no link between changes in BMI or weight gain and COC or SP. Individuals receiving COC had a higher BMI after 12 months of treatment than those on SPIOMET. It is, however, still controversial. Females with a baseline depressed mood reported perceived weight changes in a research done by Hall et al. [29].

Side Effects of Spironolactone and COC

In a WHO-led study, COC was linked to an increased relative risk of VTE. It was also revealed that high doses of ethinyl estradiol were associated with a considerably elevated risk of thrombotic stroke and myocardial infarction. According to a comprehensive review of observational data, third-generation COCs are associated with a greater incidence of VTE than second-generation COCs. There is a significant association between COC use and the risk of hypertension.

Spironolactone is useful in patients with documented cardiovascular disease and heart failure, according to Anand et al. [46]. SP medication was demonstrated to lower triglycerides, elevate HDL, and lower IR, as well as normalize endothelial dysfunction, although there were no cardiovascular risk benefits when combined with a COC.

Women who took COC for more than eight years had a statistically significantly higher chance of developing any cancer (adjusted relative risk 1.22, 1.07 to 1.39). Increased rates of cervical, central nervous system, or pituitary cancer and decreased chances of the uterine body and ovarian cancer were all associated with longer durations of oral contraceptive usage.

There are concerns about the COC's compliance and continuance. Participants with depressed moods were more than twice as likely to report the difference in mood and body weight changes. Young women who had perceived weight changes were less likely to maintain their COC compliance.

Miscellaneous

Anand et al.'s study showed that Spironolactone can help persons with heart disease and heart failure [46].

Limitations

Among the limitations of our investigation, we accept the scarcity of clinical studies in the treatment of adolescent PCOS with Spironolactone. Given the high incidence of PCOS, there is future potential for clinical studies (i) Adolescent PCOS management can be improved by employing a combination of drugs rather than a mono-therapy with current standard treatment options. (ii) Similar to previous systematic reviews of adolescent PCOS, most studies have a limited sample size for adolescent PCOS as it is most difficult to diagnose in an adolescent population due to its overlap with pubertal features. (iii) Variably reported menstrual regulation, the use of various acne scores, and the use of varying reporting of adverse events pose a challenge.

## Conclusions

In conclusion, we are one of the first few systematic reviews to indicate that spironolactone (SP), alone or in combination, has a considerably better safety profile than combined oral contraceptives (COC). COC, on the other hand, alleviates a broader variety of PCOS symptoms, such as acne and menstrual dysregulation, as well as giving contraceptive benefits not found with SP alone. No correlation between BMI changes and COC or SP has been observed. SP mono-therapy, on the other hand, is cardioprotective, with a favorable lipid profile and a reduction in hirsutism and total testosterone levels without exacerbating dysglycemia. SP in combination with COC or metformin surpasses COC in treating menstrual dysregulation, lowering free testosterone levels with a favorable reduction in hirsutism.

SPIOMET is more effective than COC at lowering hirsutism, BMI, insulin resistance, dysglycemia, total testosterone level, and menstrual regulation without increasing serum HDL. Long-term COC use has been related to an increased risk of women acquiring venous thromboembolism, hypertension, dyslipidemia, an increase in LDL, dysglycemia, and a higher risk of cervical, central nervous system, or pituitary cancer. Young women who experienced perceived weight changes were less likely to sustain their COC compliance. Given the widespread prevalence of COC usage, the significance of rigorous monitoring of women who use COC should be emphasized.

## References

[1] Escobar-Morreale HF (2018). Polycystic ovary syndrome: definition, aetiology, diagnosis and treatment. Nat Rev Endocrinol.

[2] Azziz R, Carmina E, Dewailly D (2006). Positions statement: criteria for defining polycystic ovary syndrome as a predominantly hyperandrogenic syndrome: an Androgen Excess Society guideline. J Clin Endocrinol Metab.

[3] Ndefo UA, Eaton A, Green MR (2013). Polycystic ovary syndrome: a review of treatment options with a focus on pharmacological approaches. P T.

[4] Bozdag G, Mumusoglu S, Zengin D, Karabulut E, Yildiz BO (2016). The prevalence and phenotypic features of polycystic ovary syndrome: a systematic review and meta-analysis. Hum Reprod.

[5] McGowan MP (2011). Polycystic ovary syndrome: a common endocrine disorder and risk factor for vascular disease. Curr Treat Options Cardiovasc Med.

[6] Peña AS, Witchel SF, Hoeger KM (2020). Adolescent polycystic ovary syndrome according to the international evidence-based guideline. BMC Med.

[7] Broekmans FJ, Knauff EA, Valkenburg O, Laven JS, Eijkemans MJ, Fauser BC (2006). PCOS according to the Rotterdam consensus criteria: change in prevalence among WHO-II anovulation and association with metabolic factors. BJOG.

[8] Sharma A, Atiomo W (2003). Recent developments in polycystic ovary syndrome. Curr Obstet Gynecol Rep.

[9] Daniilidis A, Dinas K (2009). Long term health consequences of polycystic ovarian syndrome: a review analysis. Hippokratia.

[10] Amiri M, Golsorkhtabaramiri M, Esmaeilzadeh S, Ghofrani F, Bijani A, Ghorbani L, Delavar MA (2014). Effect of metformin and flutamide on anthropometric indices and laboratory tests in obese/overweight PCOS women under hypocaloric diet. J Reprod Infertil.

[11] Gambineri A, Patton L, Vaccina A (2006). Treatment with flutamide, metformin, and their combination added to a hypocaloric diet in overweight-obese women with polycystic ovary syndrome: a randomized, 12-month, placebo-controlled study. J Clin Endocrinol Metab.

[12] Ganie MA, Khurana ML, Eunice M, Gupta N, Gulati M, Dwivedi SN, Ammini AC (2004). Comparison of efficacy of spironolactone with metformin in the management of polycystic ovary syndrome: an open-labeled study. J Clin Endocrinol Metab.

[13] Tartagni M, Schonauer MM, Cicinelli E, Petruzzelli F, De Pergola G, De Salvia MA, Loverro G (2004). Intermittent low-dose finasteride is as effective as daily administration for the treatment of hirsute women. Fertil Steril.

[14] Falsetti L, Gambera A, Legrenzi L, Iacobello C, Bugari G (1999). Comparison of finasteride versus flutamide in the treatment of hirsutism. Eur J Endocrinol.

[15] Shah D, Patil M (2018). Consensus statement on the use of oral contraceptive pills in polycystic ovarian syndrome women in India. J Hum Reprod Sci.

[16] Balen AH (2017). Polycystic ovary syndrome (PCOS). Obstet. Gynecol.

[17] Zulian E, Sartorato P, Benedini S, Baro G, Armanini D, Mantero F, Scaroni C (2005). Spironolactone in the treatment of polycystic ovary syndrome: effects on clinical features, insulin sensitivity and lipid profile. J Endocrinol Invest.

[18] Kamboj MK, Bonny AE (2017). Polycystic ovary syndrome in adolescence: diagnostic and therapeutic strategies. Transl Pediatr.

[19] Hannaford PC, Iversen L, Macfarlane TV, Elliott AM, Angus V, Lee AJ (2010). Mortality among contraceptive pill users: cohort evidence from Royal College of General Practitioners' Oral Contraception Study. BMJ.

[20] Liberati A, Altman DG, Tetzlaff J (2009). The PRISMA statement for reporting systematic reviews and meta-analyses of studies that evaluate healthcare interventions: explanation and elaboration. BMJ.

[21] Moher D, Liberati A, Tetzlaff J, Altman DG (2009). Preferred reporting items for systematic reviews and meta-analyses: the PRISMA statement. PLoS Med.

[22] Page MJ, McKenzie JE, Bossuyt PM (2021). The PRISMA 2020 statement: an updated guideline for reporting systematic reviews. BMJ.

[23] Sawyer SM, Azzopardi PS, Wickremarathne D, Patton GC (2018). The age of adolescence. Lancet Child Adolesc Health.

[24] (2004). Revised 2003 consensus on diagnostic criteria and long-term health risks related to polycystic ovary syndrome. Fertil Steril.

[25] Azziz R, Carmina E, Dewailly D (2009). The Androgen Excess and PCOS Society criteria for the polycystic ovary syndrome: the complete task force report. Fertil Steril.

[26] Shea BJ, Reeves BC, Wells G (2017). AMSTAR 2: a critical appraisal tool for systematic reviews that include randomised or non-randomised studies of healthcare interventions, or both. BMJ.

[27] Baethge C, Goldbeck-Wood S, Mertens S (2019). SANRA-a scale for the quality assessment of narrative review articles. Res Integr Peer Rev.

[28] Higgins JP, Altman DG, Gøtzsche PC (2011). The Cochrane Collaboration's tool for assessing risk of bias in randomised trials. BMJ.

[29] Hall KS, White KO, Rickert VI, Reame N, Westhoff C (2012). Influence of depressed mood and psychological stress symptoms on perceived oral contraceptive side effects and discontinuation in young minority women. Contraception.

[30] Domecq JP, Prutsky G, Mullan RJ (2013). Adverse effects of the common treatments for polycystic ovary syndrome: a systematic review and meta-analysis. J Clin Endocrinol Metab.

[31] Alpañés M, Álvarez-Blasco F, Fernández-Durán E, Luque-Ramírez M, Escobar-Morreale HF (2017). Combined oral contraceptives plus spironolactone compared with metformin in women with polycystic ovary syndrome: a one-year randomized clinical trial. Eur J Endocrinol.

[32] Liu H, Yao J, Wang W, Zhang D (2017). Association between duration of oral contraceptive use and risk of hypertension: a meta-analysis. J Clin Hypertens (Greenwich).

[33] Díaz M, Gallego-Escuredo JM, López-Bermejo A, de Zegher F, Villarroya F, Ibáñez L (2018). Low-dose spironolactone-pioglitazone-metformin normalizes circulating fetuin-a concentrations in adolescent girls with polycystic ovary syndrome. Int J Endocrinol.

[34] Alalami H, Sathyapalan T, Atkin SL (2018). Cardiovascular profile of pharmacological agents used for the management of polycystic ovary syndrome. Ther Adv Endocrinol Metab.

[35] Almalki HH, Alshibani TM, Alhifany AA, Almohammed OA (2020). Comparative efficacy of statins, metformin, spironolactone and combined oral contraceptives in reducing testosterone levels in women with polycystic ovary syndrome: a network meta-analysis of randomized clinical trials. BMC Womens Health.

[36] Ibáñez L, Díaz M, García-Beltrán C, Malpique R, Garde E, López-Bermejo A, de Zegher F (2020). Toward a treatment normalizing ovulation rate in adolescent girls with polycystic ovary syndrome. J Endocr Soc.

[37] Al Khalifah RA, Florez ID, Zoratti MJ, Dennis B, Thabane L, Bassilious E (2021). Efficacy of treatments for polycystic ovarian syndrome management in adolescents. J Endocr Soc.

[38] Al Khalifah RA, Florez ID, Dennis B, Thabane L, Bassilious E (2016). Metformin or oral contraceptives for adolescents with polycystic ovarian syndrome: a meta-analysis. Pediatrics.

[39] Stefan N, Hennige AM, Staiger H (2006). Alpha2-Heremans-Schmid glycoprotein/fetuin-A is associated with insulin resistance and fat accumulation in the liver in humans. Diabetes Care.

[40] Ix JH, Shlipak MG, Brandenburg VM, Ali S, Ketteler M, Whooley MA (2006). Association between human fetuin-A and the metabolic syndrome: data from the Heart and Soul Study. Circulation.

[41] Sindhu S, Akhter N, Shenouda S, Wilson A, Ahmad R (2016). Plasma fetuin-A/α2-HS-glycoprotein correlates negatively with inflammatory cytokines, chemokines and activation biomarkers in individuals with type-2 diabetes. BMC Immunol.

[42] Lidegaard Ø, Løkkegaard E, Jensen A, Skovlund CW, Keiding N (2012). Thrombotic stroke and myocardial infarction with hormonal contraception. N Engl J Med.

[43] Kemmeren JM, Algra A, Grobbee DE (2001). Third generation oral contraceptives and risk of venous thrombosis: meta-analysis. BMJ.

[44] Ageno W, Becattini C, Brighton T, Selby R, Kamphuisen PW (2008). Cardiovascular risk factors and venous thromboembolism: a meta-analysis. Circulation.

[45] Dokras A (2016). Noncontraceptive use of oral combined hormonal contraceptives in polycystic ovary syndrome-risks versus benefits. Fertil Steril.

[46] Anand IS, Claggett B, Liu J (2017). Interaction between spironolactone and natriuretic peptides in patients with heart failure and preserved ejection fraction: from the TOPCAT trial. JACC Heart Fail.

[47] Bird ST, Hartzema AG, Brophy JM, Etminan M, Delaney JA (2013). Risk of venous thromboembolism in women with polycystic ovary syndrome: a population-based matched cohort analysis. CMAJ.

[48] Sathyapalan T, Atkin SL (2012). Mechanisms in endocrinology: recent advances in cardiovascular aspects of polycystic ovary syndrome. Eur J Endocrinol.

